# Supplementary material for: Nanoparticles destabilizing the cell membranes triggered by NIR light for cancer imaging and photo-immunotherapy
Source: Nat Commun. 2024 Jul 17;15:6026. doi: 10.1038/s41467-024-50020-w (PMC11255282; doi:10.1038/s41467-024-50020-w)
Supplement: Supplementary file 1 — Supplementary Information [file 41467_2024_50020_MOESM1_ESM.pdf]

Supporting Information

Nanoparticles Destabilizing the Cell Membranes Triggered by NIR Light for Cancer Imaging and Photo-Immunotherapy

Dongsheng Tang[1,2], *Minhui Cui*[1,2], *Bin Wang*[1,2], *Ganghao Liang*[1,2], *Hanchen Zhang*[1,2], *Haihua Xiao*[1,2]*

[1]Beijing National Laboratory for Molecular Sciences, Laboratory of Polymer Physics and Chemistry, Institute of Chemistry, Chinese Academy of Sciences, Beijing, 100190, P. R. China

[2]University of Chinese Academy of Sciences, Beijing 100049, P. R. China

E-mail: hhxiao@iccas.ac.cn

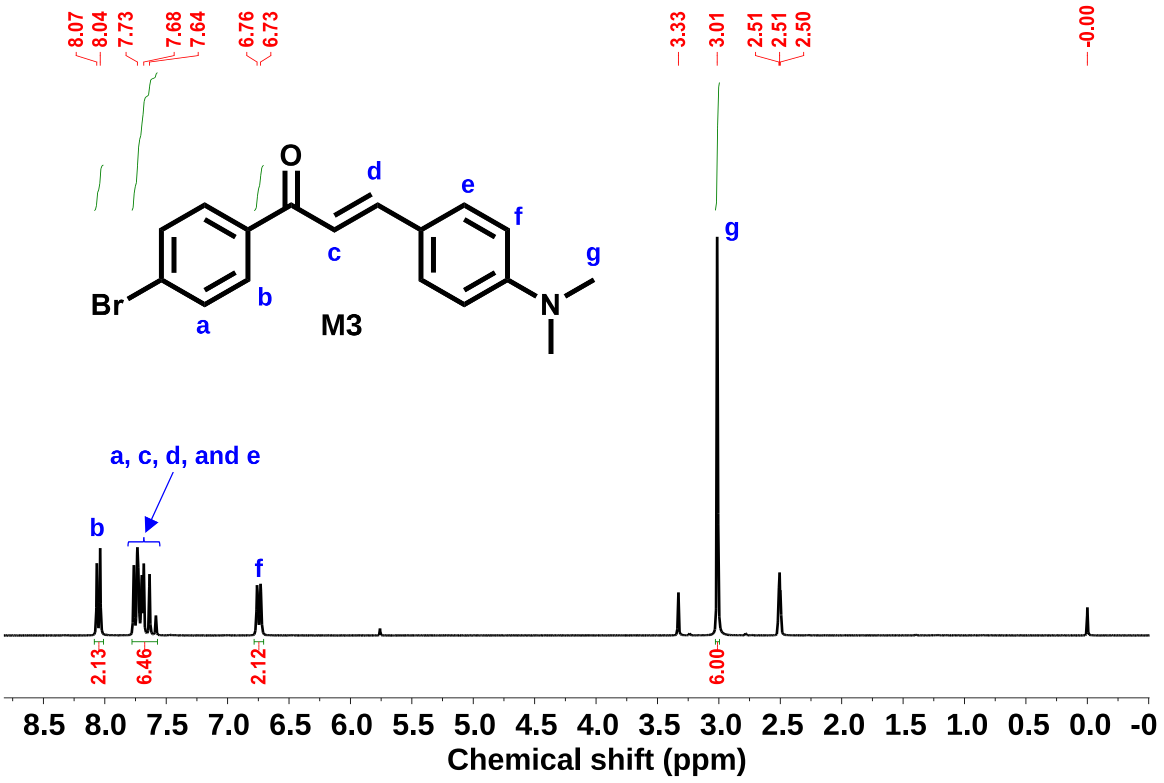

Supplementary Fig. 1. ^{1}H NMR spectrum of M3 in DMSO-*d*6. ^{1}H NMR chemical shift was assigned (a-g).

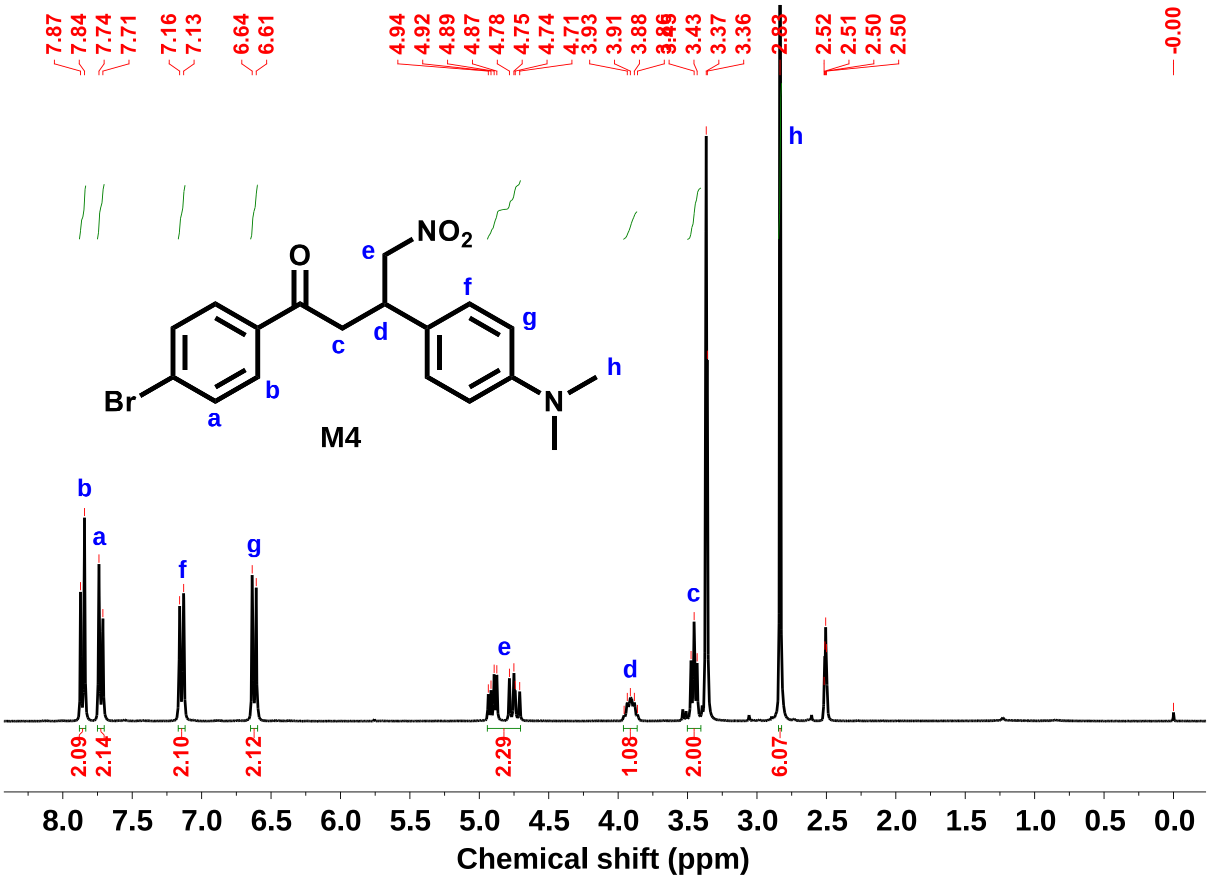

Supplementary Fig. 2. ^{1}H NMR spectrum of M4 in DMSO-*d*6. ^{1}H NMR chemical shift was assigned (a-h).

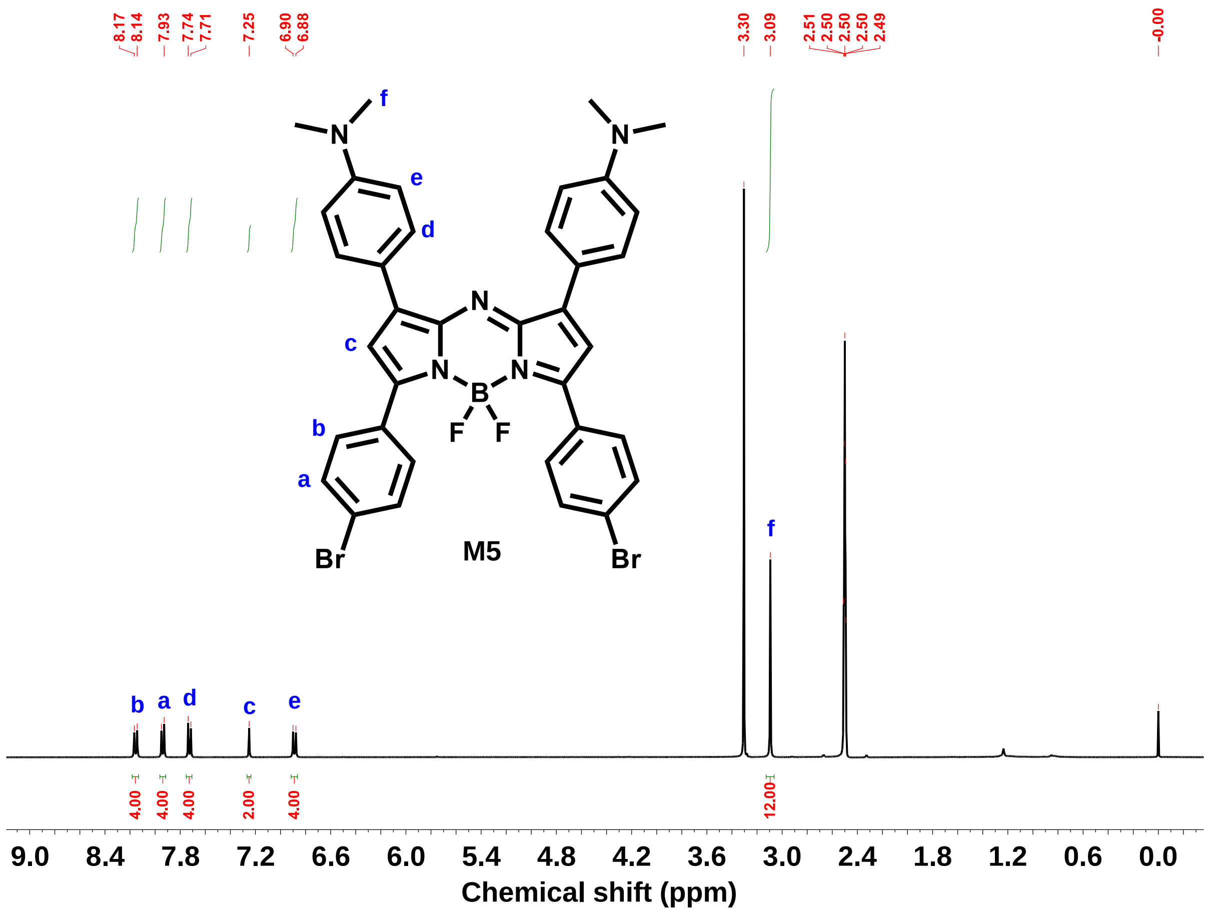

Supplementary Fig. 3. ^{1}H NMR spectrum of M5 in DMSO-*d*6. ^{1}H NMR chemical shift was assigned (a-f).

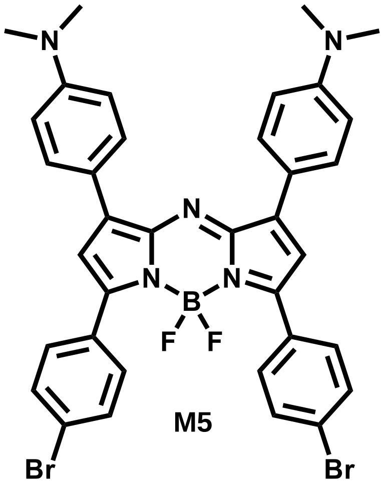

Chemical Formula: $C_{36}H_{30}BBr_2F_2N_5$

Calculation: $[M]^+$ m/z 741.091

Found: $[M]^+$ m/z 741.376

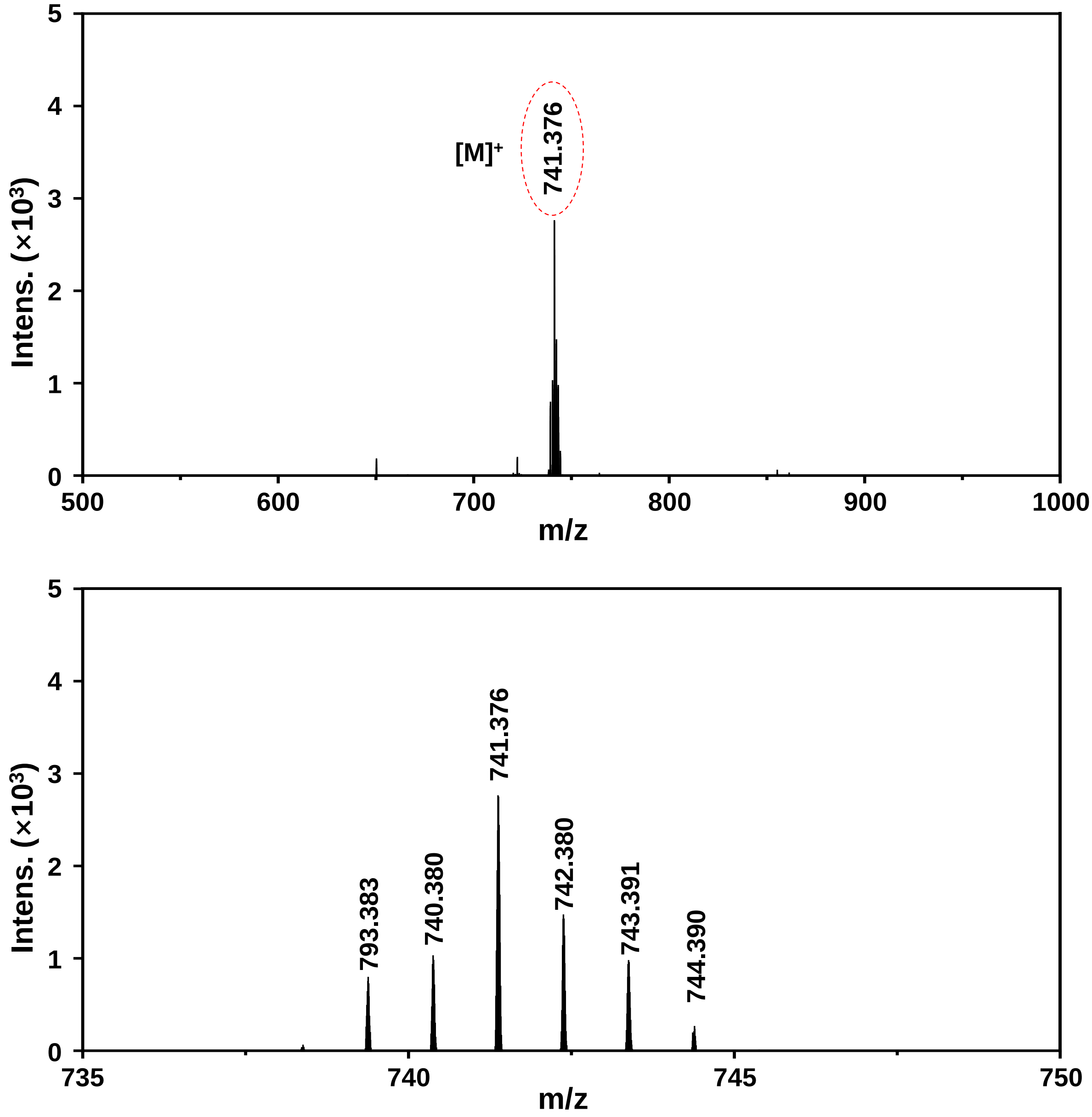

Supplementary Fig. 4. MALDI-TOF-MS spectrum of M5.

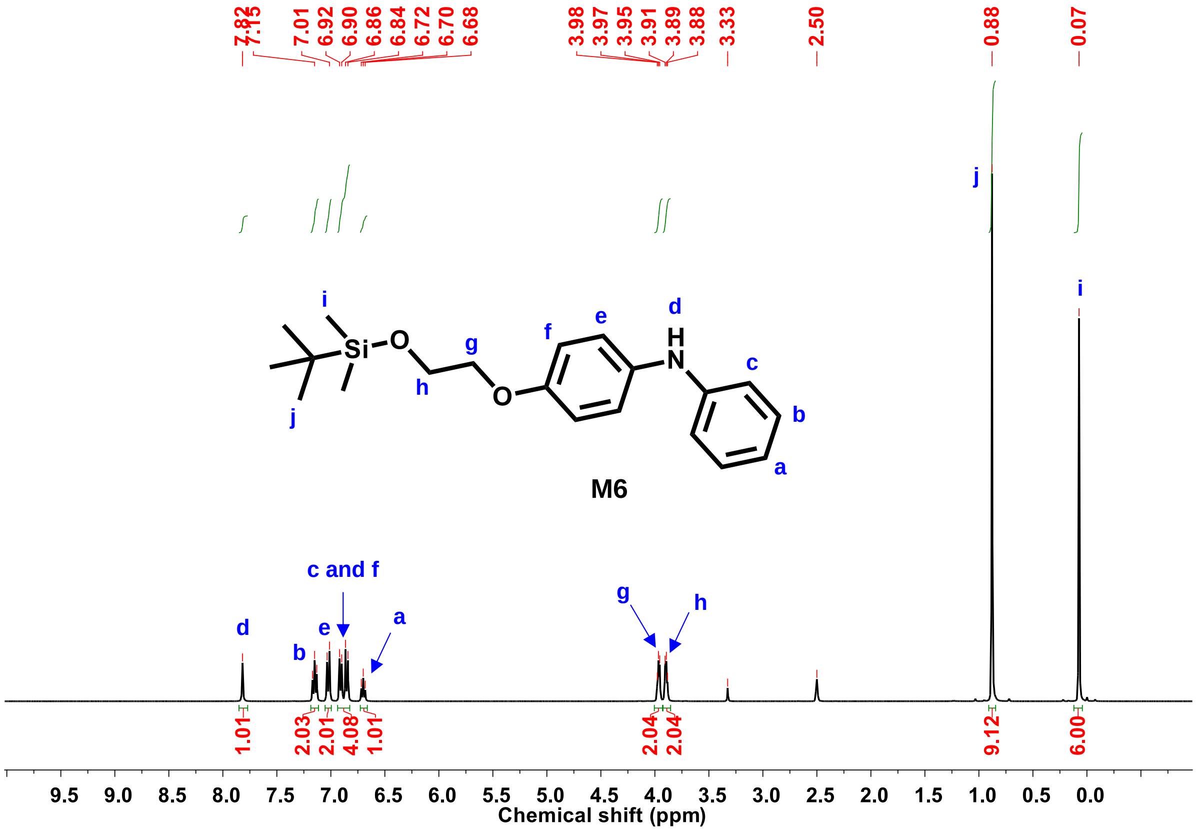

Supplementary Fig. 5. ^{1}H NMR spectrum of M6 in DMSO-d_6. ^{1}H NMR chemical shift was assigned (a-j).

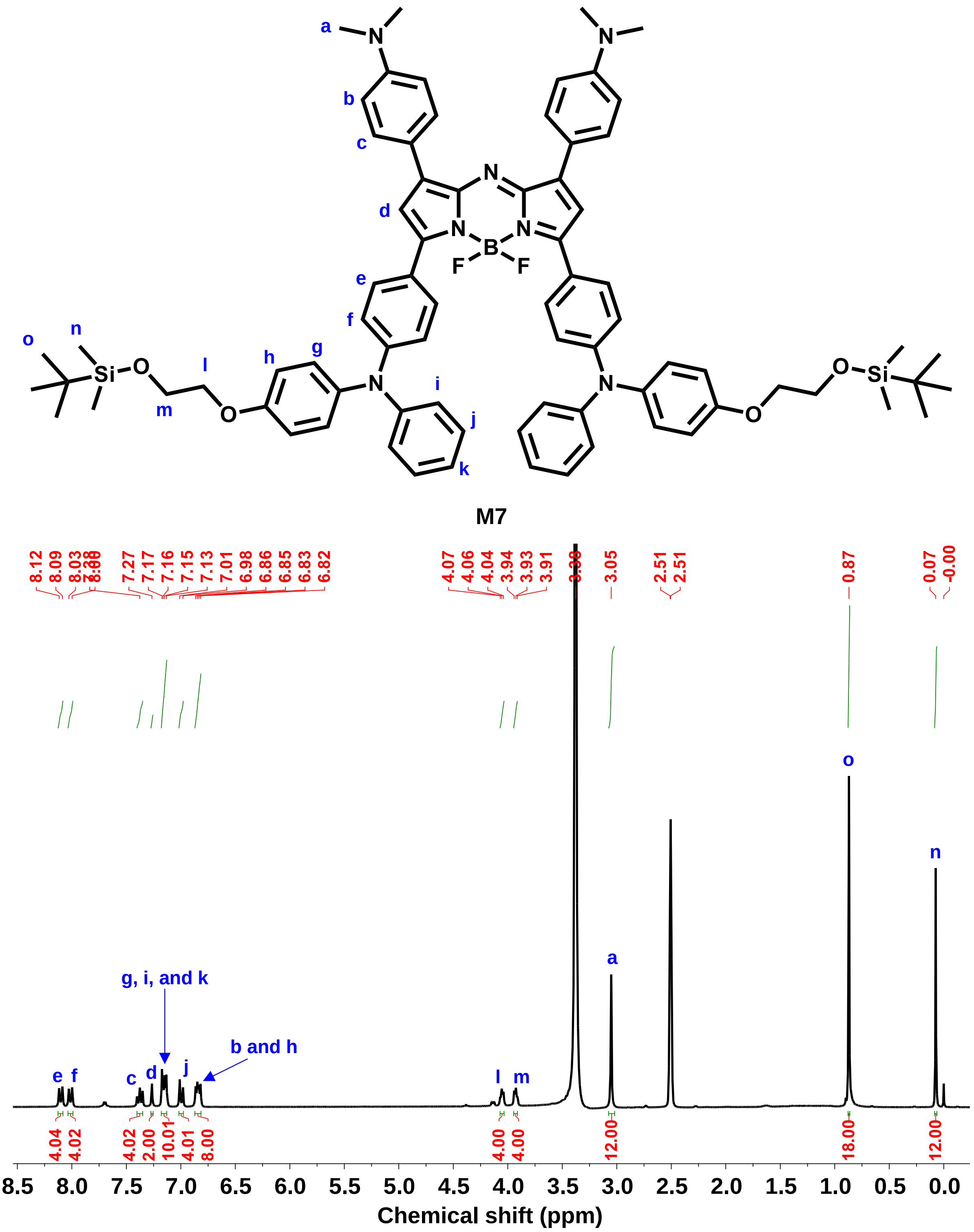

Supplementary Fig. 6. ^{1}H NMR spectrum of M7 in DMSO-d_6. ^{1}H NMR chemical shift was assigned (a-o).

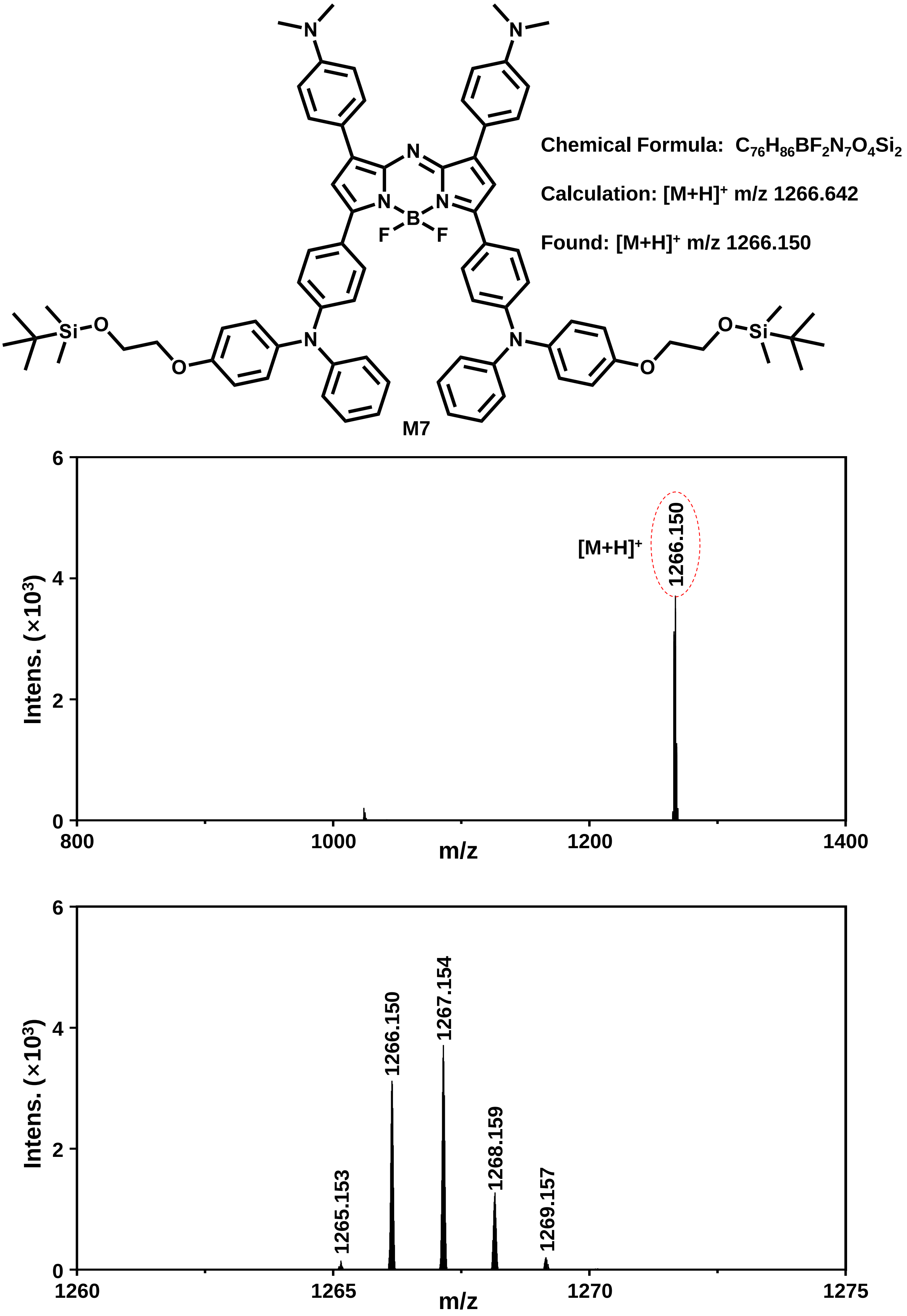

Supplementary Fig. 7. MALDI-TOF-MS spectrum of M7.

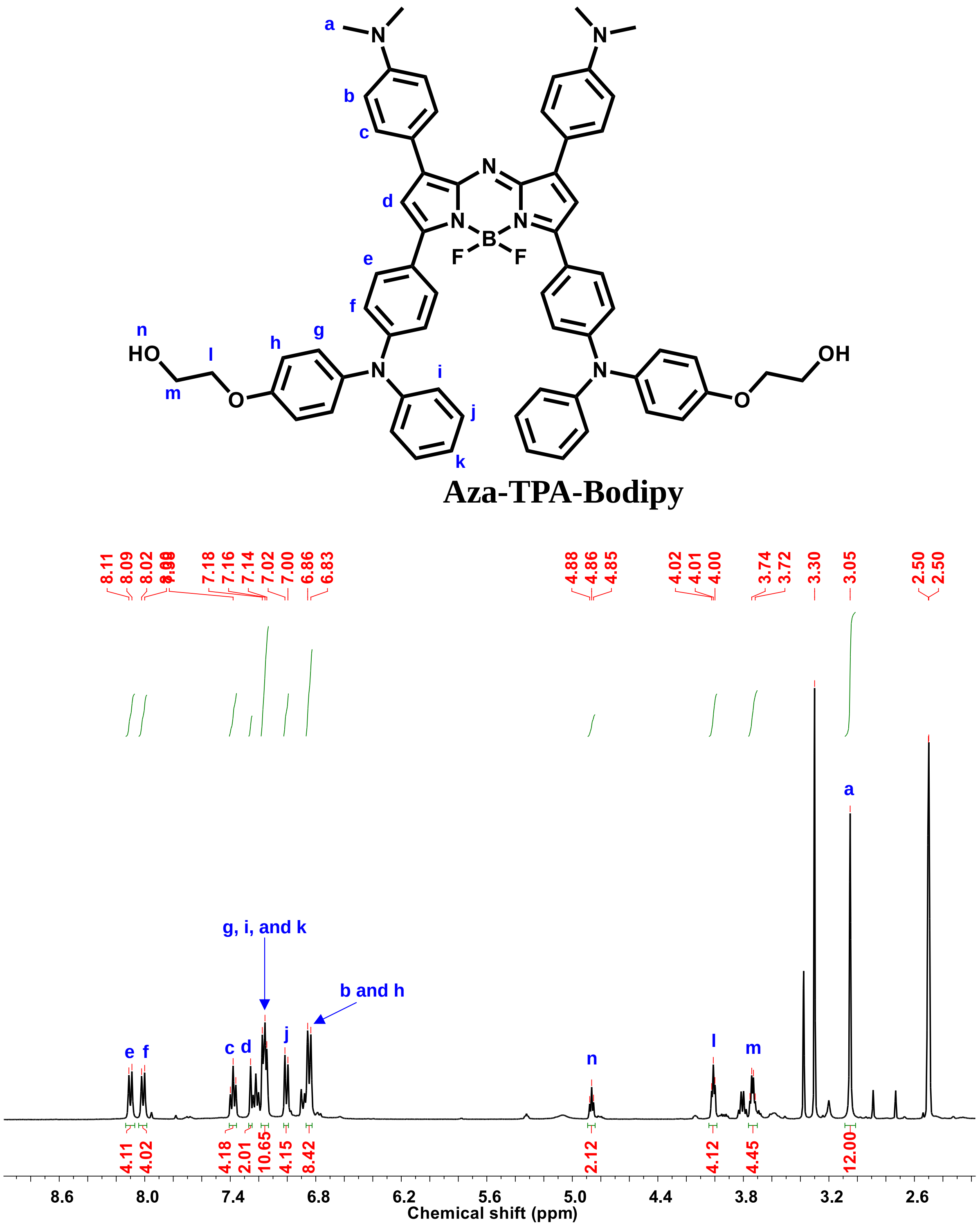

Supplementary Fig. 8. ^{1}H NMR spectrum of Aza-TPA-Bodipy in DMSO-d_6. ^{1}H NMR chemical shift was assigned (a-n).

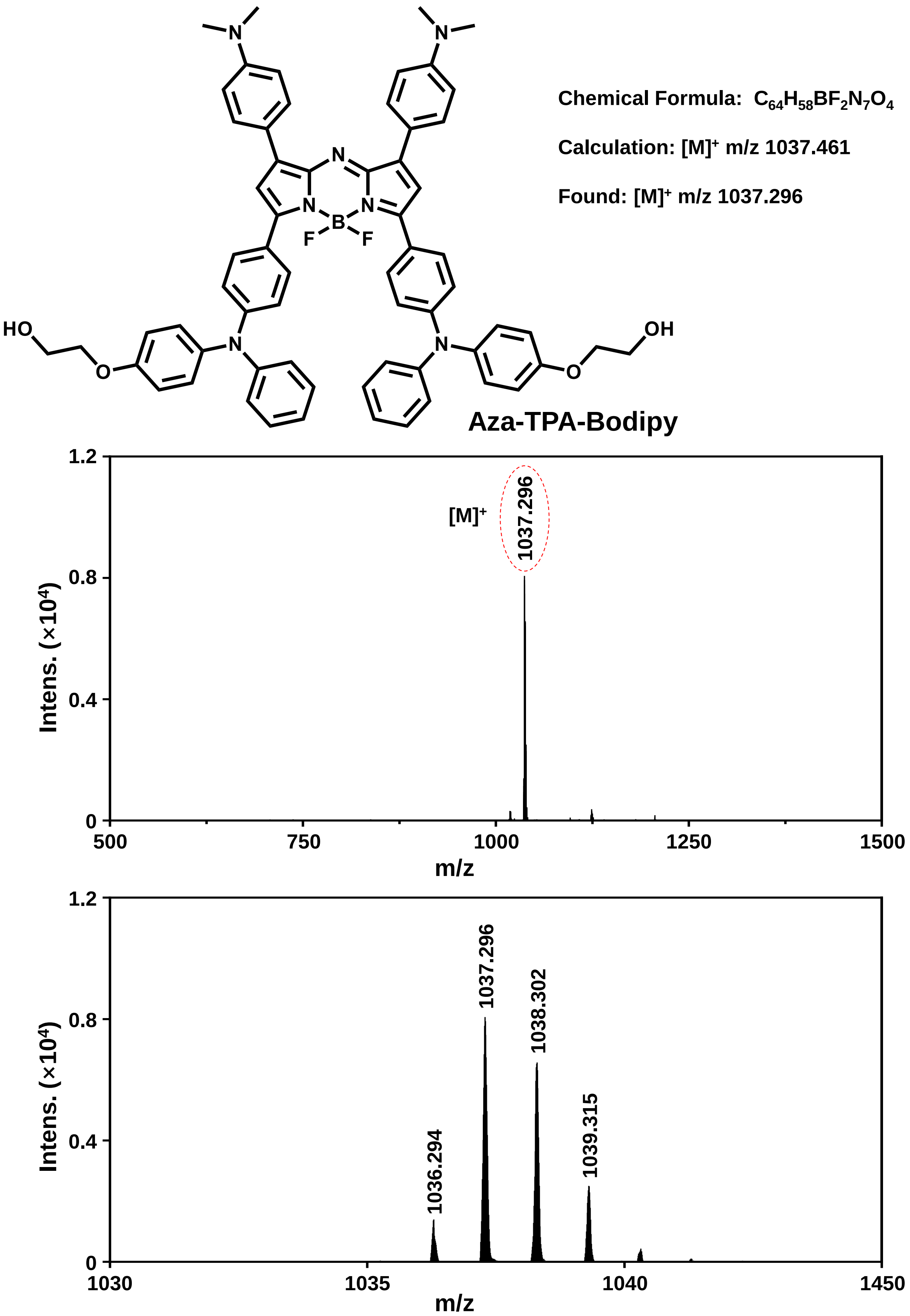

Supplementary Fig. 9. MALDI-TOF-MS spectrum of Aza-TPA-Bodipy.

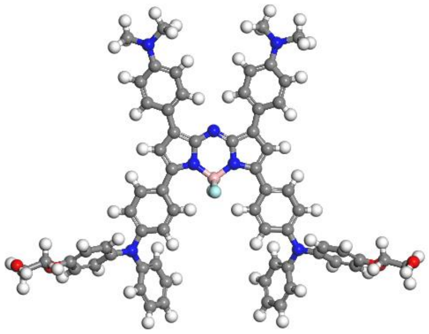

S_0 optimized geometry

Supplementary Fig. 10. The optimized structures of Aza-TPA-Bodipy.

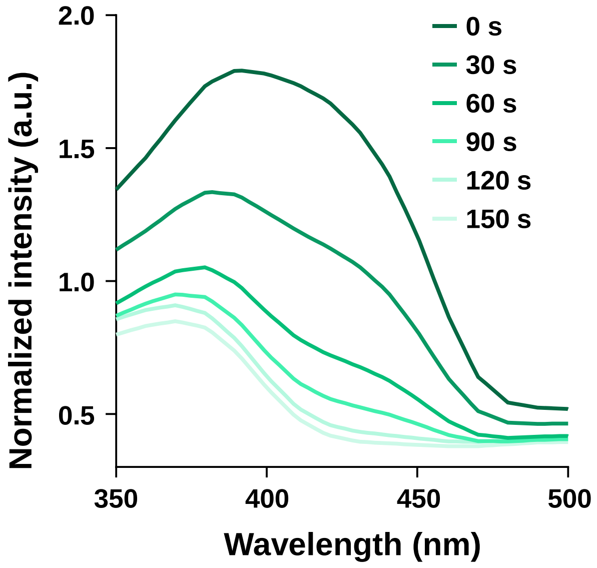

Supplementary Fig. 11. Absorbance change of Aza-TPA-Bodipy after 808 nm light irradiation (1.0 W cm^{-2}).

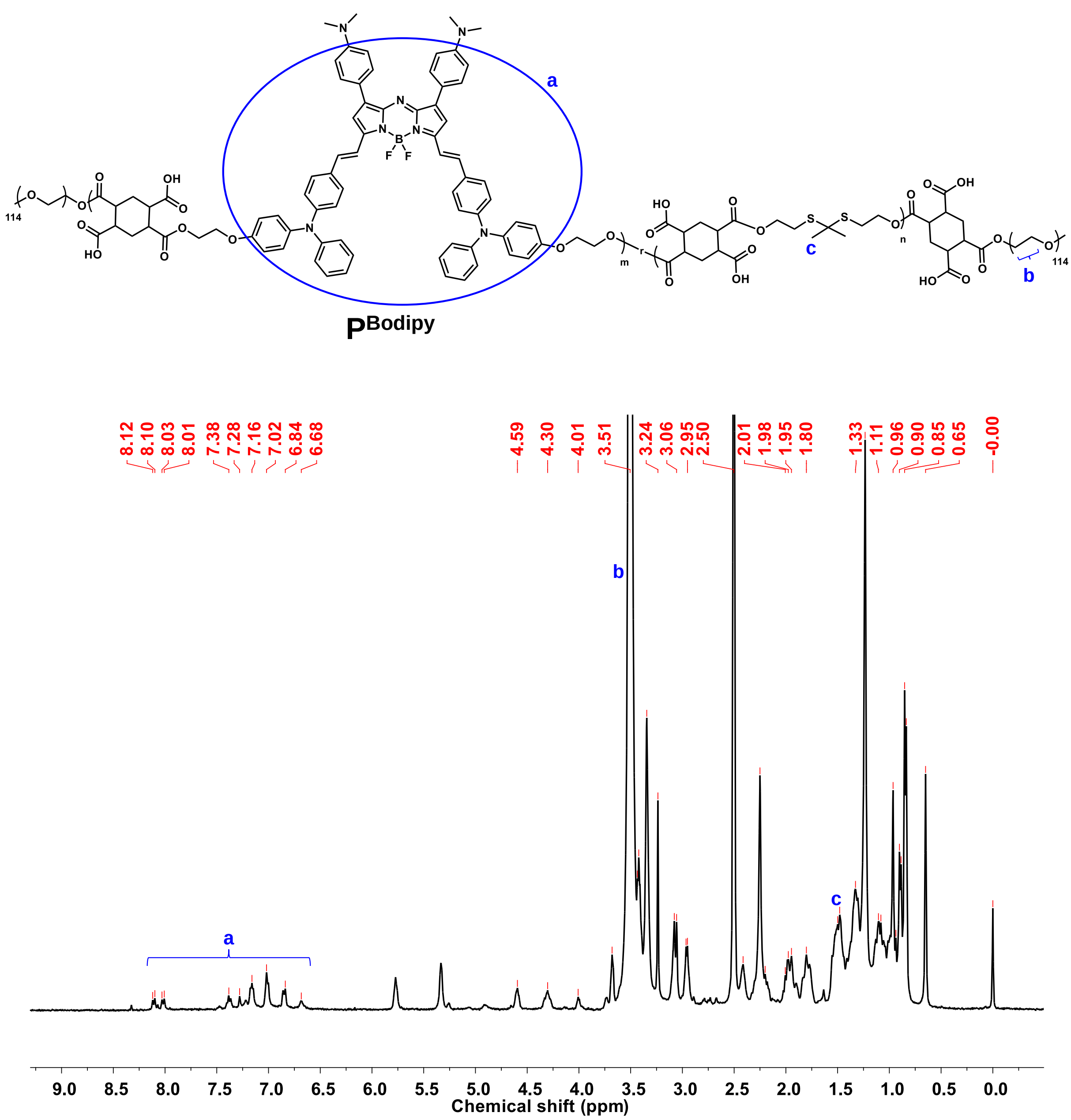

Supplementary Fig. 12. ^{1}H NMR spectrum of P^{Bodipy} in DMSO-*d6*. ^{1}H NMR chemical shift was assigned (a-c).

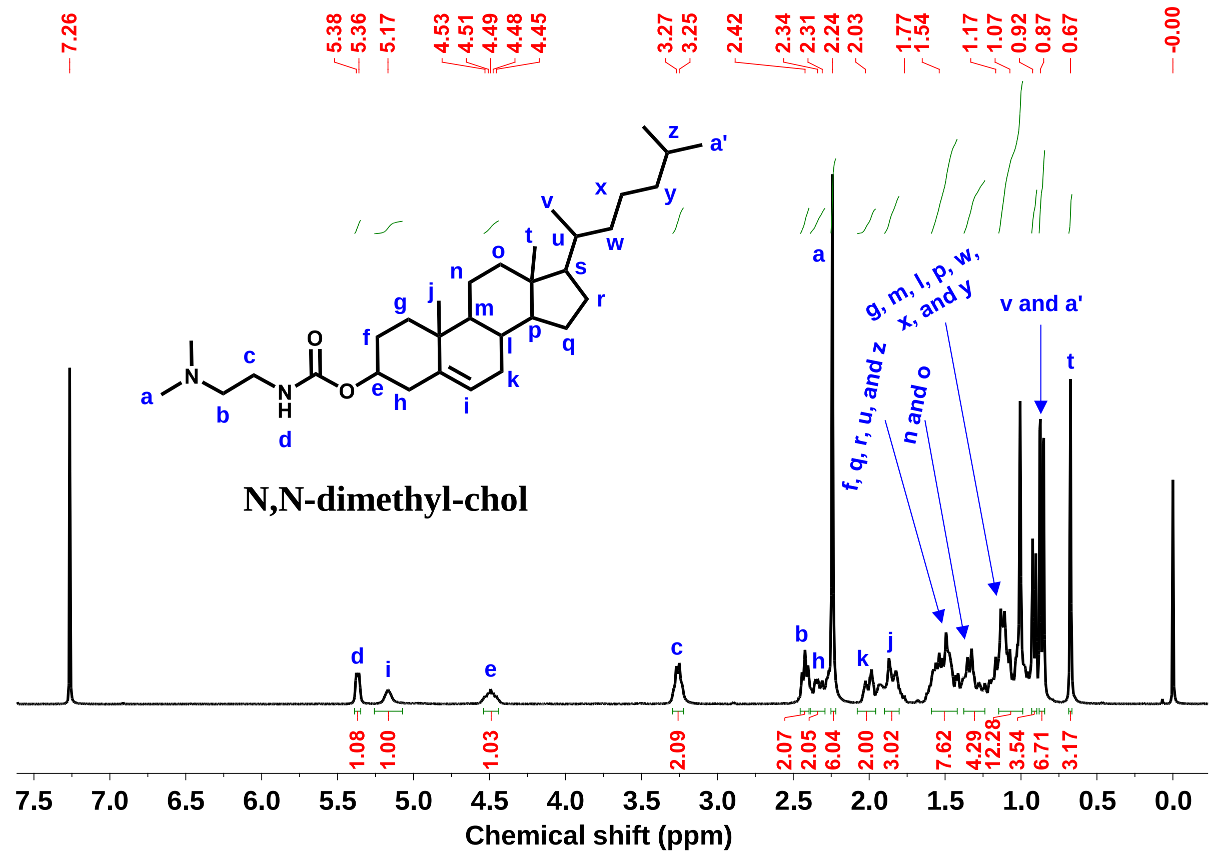

Supplementary Fig. 13. ^{1}H NMR spectrum of N,N-dimethyl-chol in $CDCl_3$. ^{1}H NMR chemical shift was assigned (a-a′).

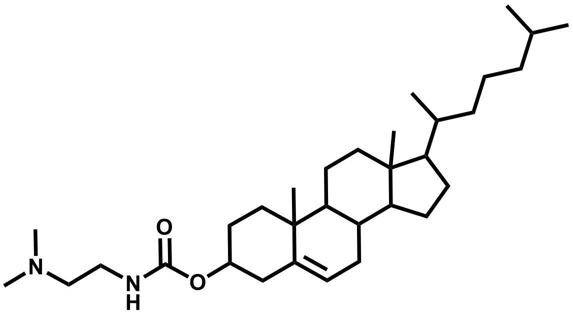

N,N-dimethyl-chol

Chemical Formula: $C_{32}H_{56}N_2O_2$

Calculation: $[M+H]^+$ m/z 501.44

Found: $[M+H]^+$ m/z 501.45

Calculation: $[M+Na]^+$ m/z 523.42

Found: $[M+Na]^+$ m/z 523.45

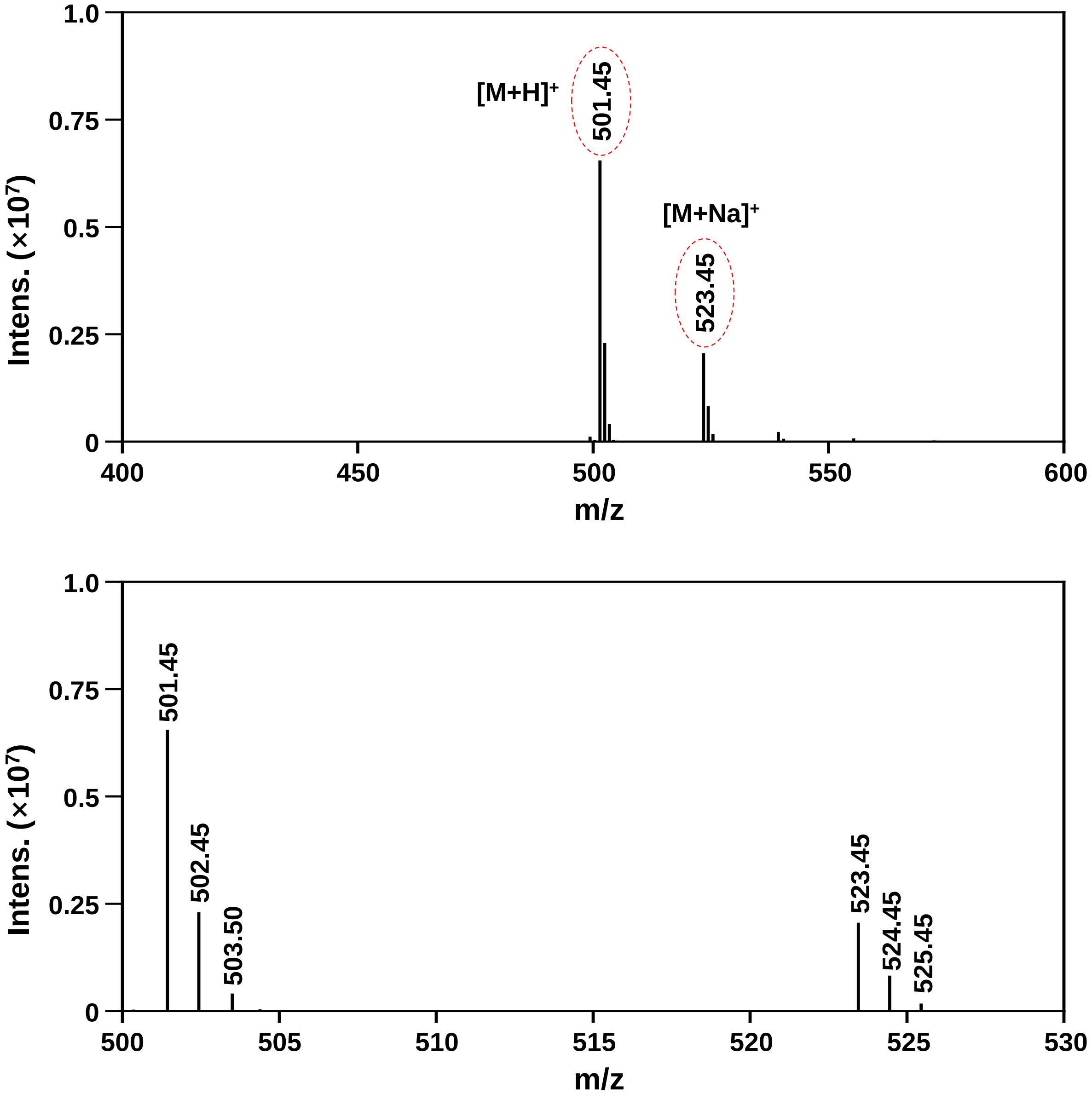

Supplementary Fig. 14. ESI-MS spectrum of N,N-dimethyl-chol.

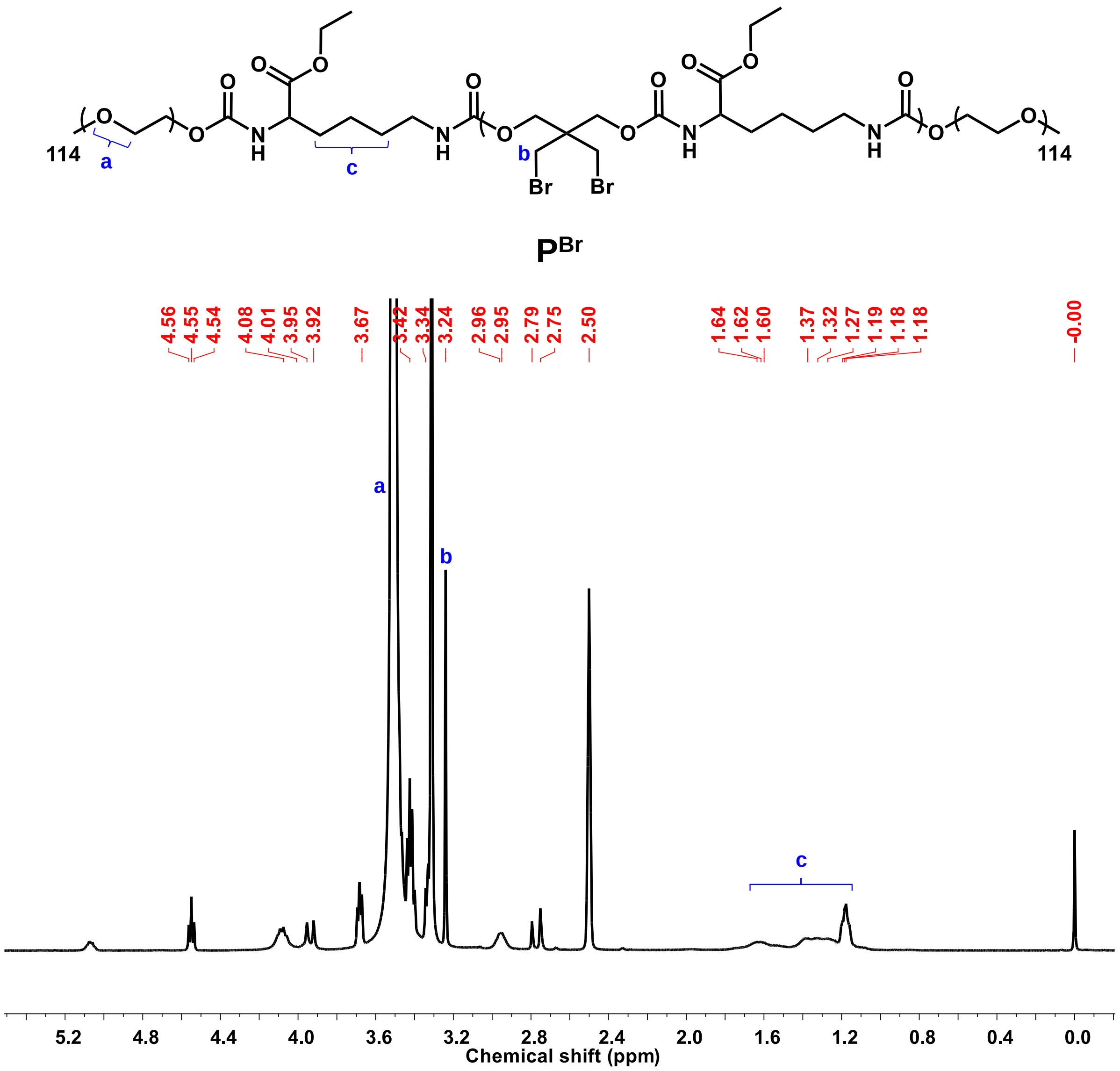

Supplementary Fig. 15. ^{1}H NMR spectrum of P^{Br} in DMSO-d_6. ^{1}H NMR chemical shift was assigned (a-c).

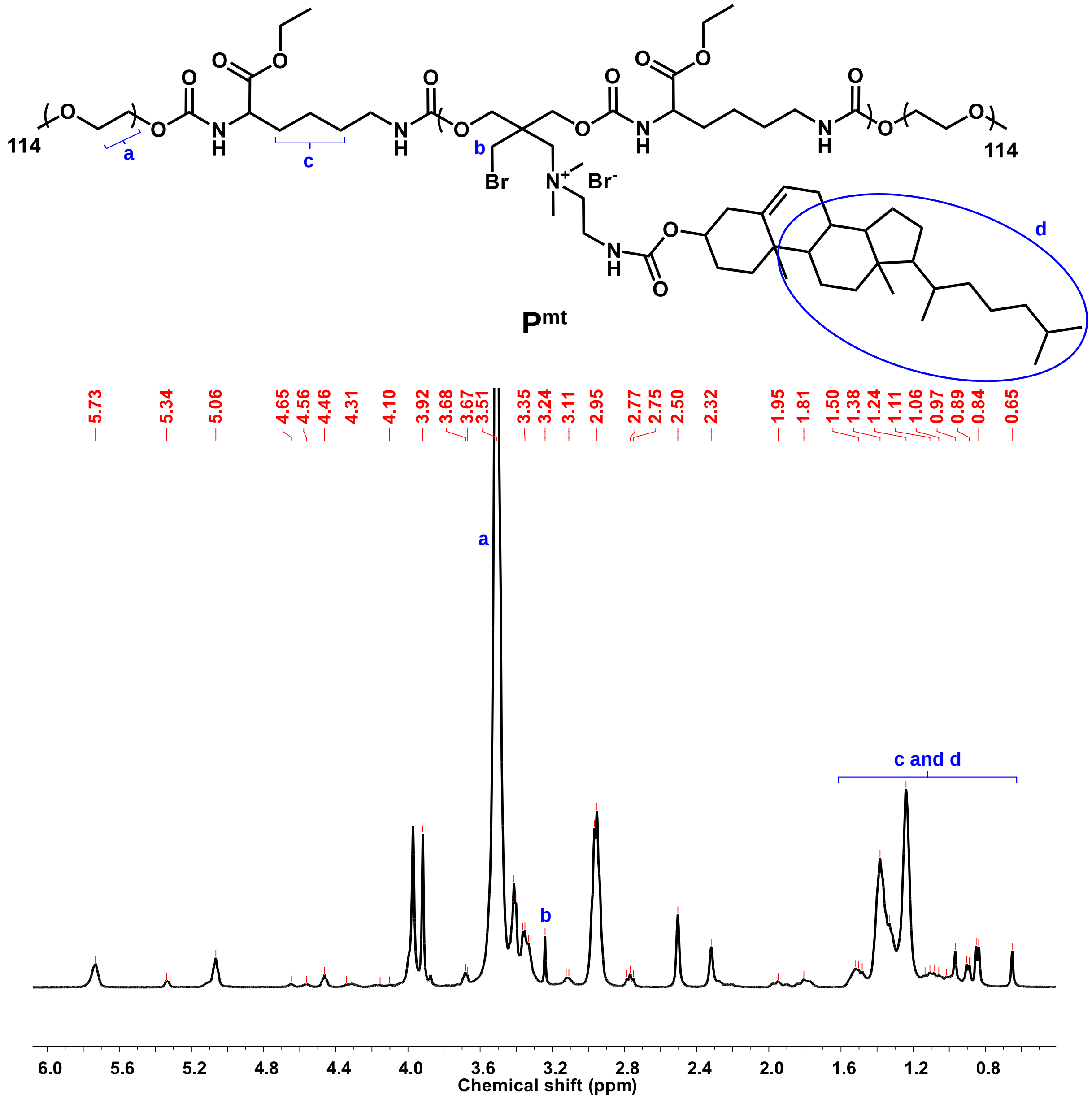

Supplementary Fig. 16. ^{1}H NMR spectrum of P^{mt} in DMSO-*d*6. ^{1}H NMR chemical shift was assigned (a-d).

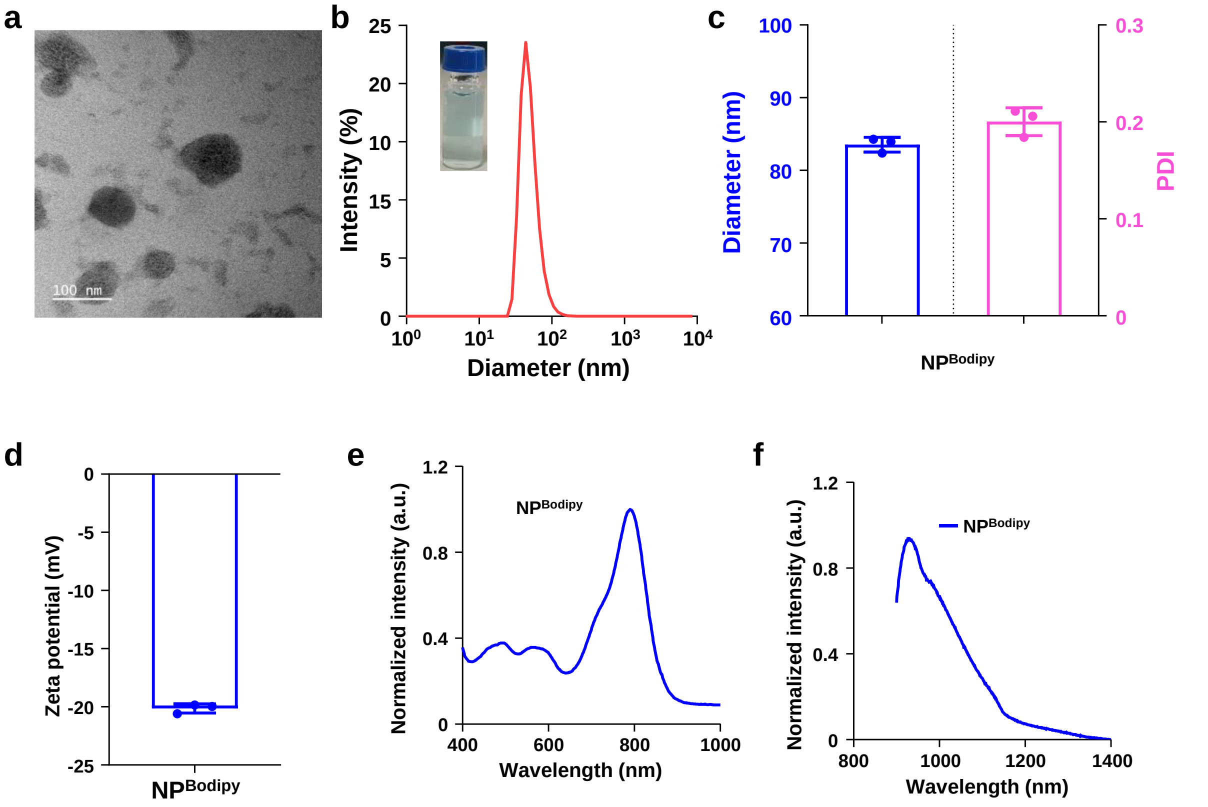

Supplementary Fig. 17. Characterization of NP^{Bodipy}. **a** The representative TEM of NP^{Bodipy} (n = 3 independent experiments). **b, c** Hydrodynamic diameters and PDI of NP^{Bodipy} (n = 3 independent samples, standard deviation for diameter and PDI are 1.02 and 0.014, respectively.). **d** Zeta potentials of mt-NP^{Bodipy} (n = 3 independent samples, standard deviation is 0.40). **e** Absorption spectra of NP^{Bodipy}. **f** PL spectra of NP^{Bodipy}. Data are presented as mean ± SD.

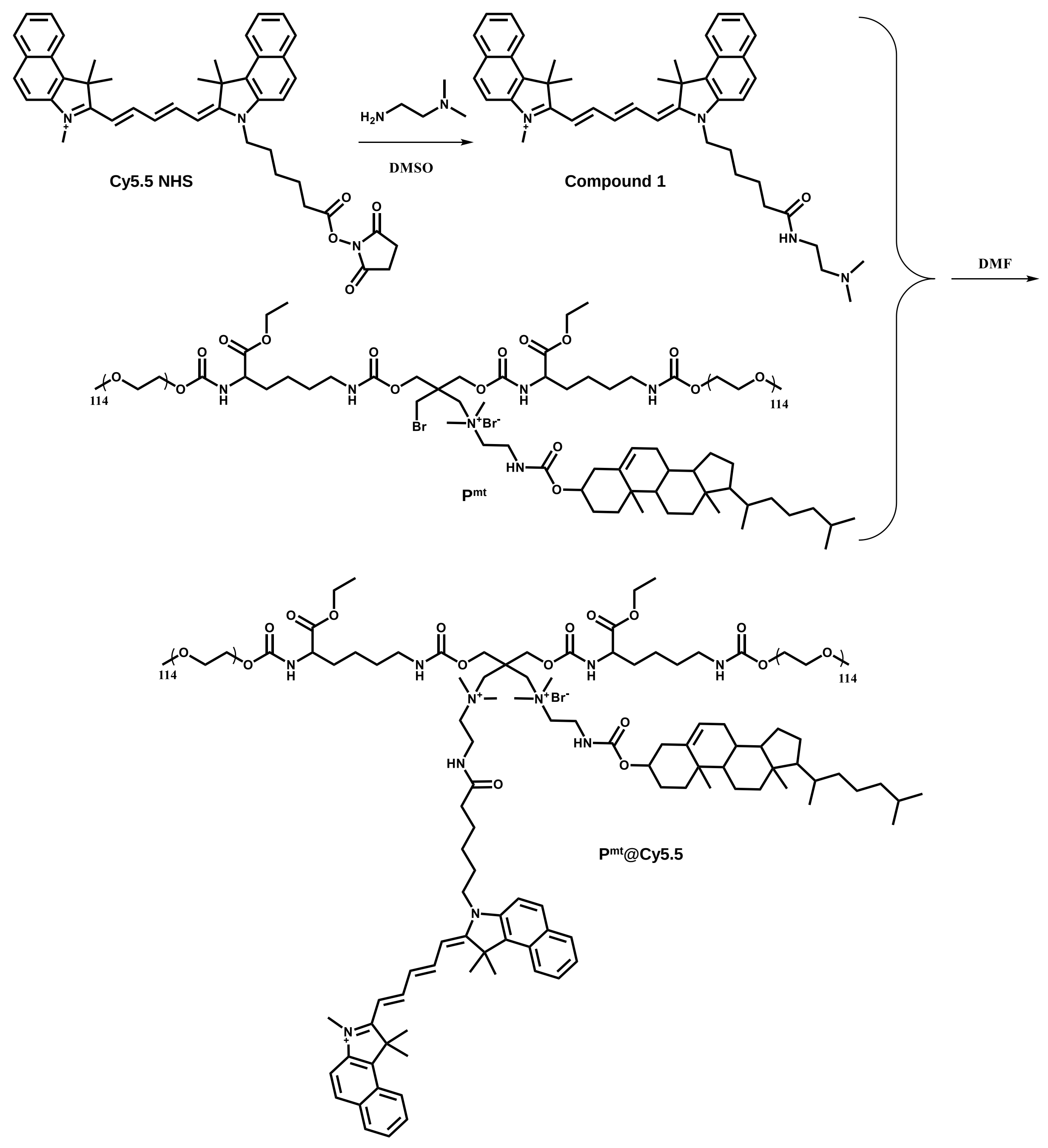

Supplementary Fig. 18. Synthetic route of P^{mt}@Cy5.5.

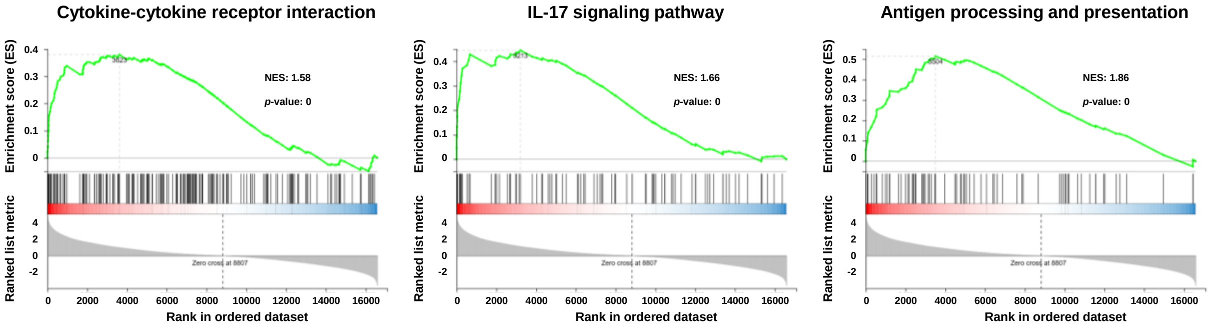

Supplementary Fig. 19. GSEA reveals positive enrichment of genes altered in mt-NP^{Bodipy} + L treatment cells.

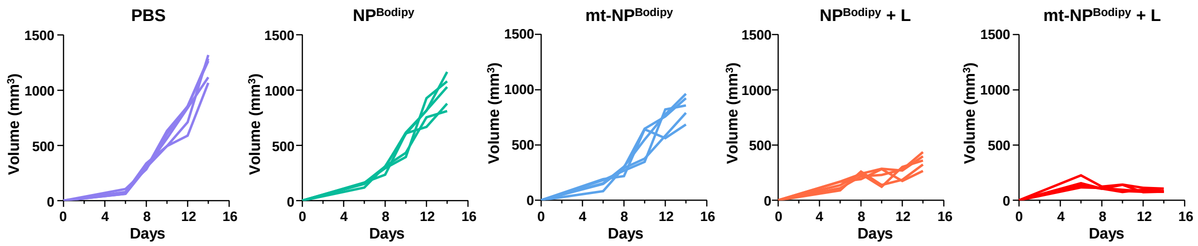

Supplementary Fig. 20. Tumor growth inhibition curves (n = 5). This is the data of tumour value each mouse in Fig. 6f, and significant differences between mt-NP^{Bodipy} + L and NP^{Bodipy} + L have been annotated in the corresponding captions (*****p* < 0.0001).

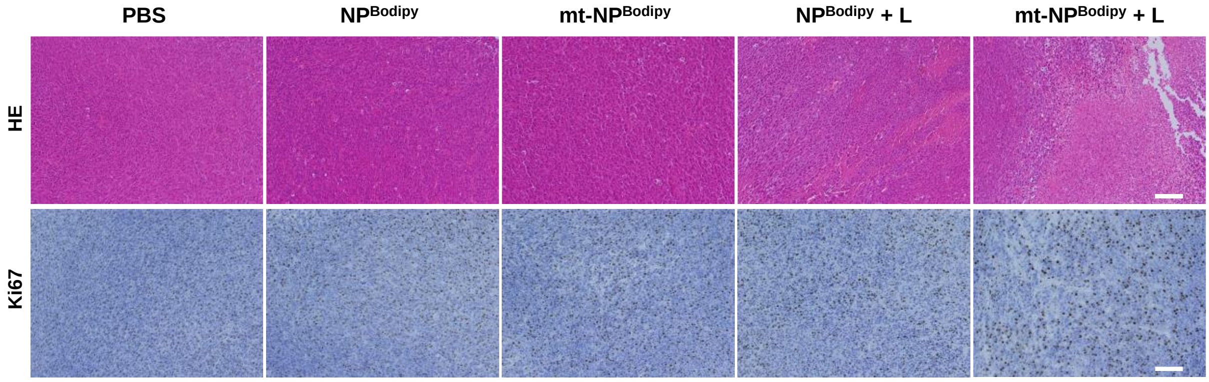

Supplementary Fig. 21. Hematoxylin and eosin (scale bar = 50 μm) and Ki67 (scale bar = 100 μm) staining of the tumor after the treatment. Experiment was repeated three times independently with similar results.

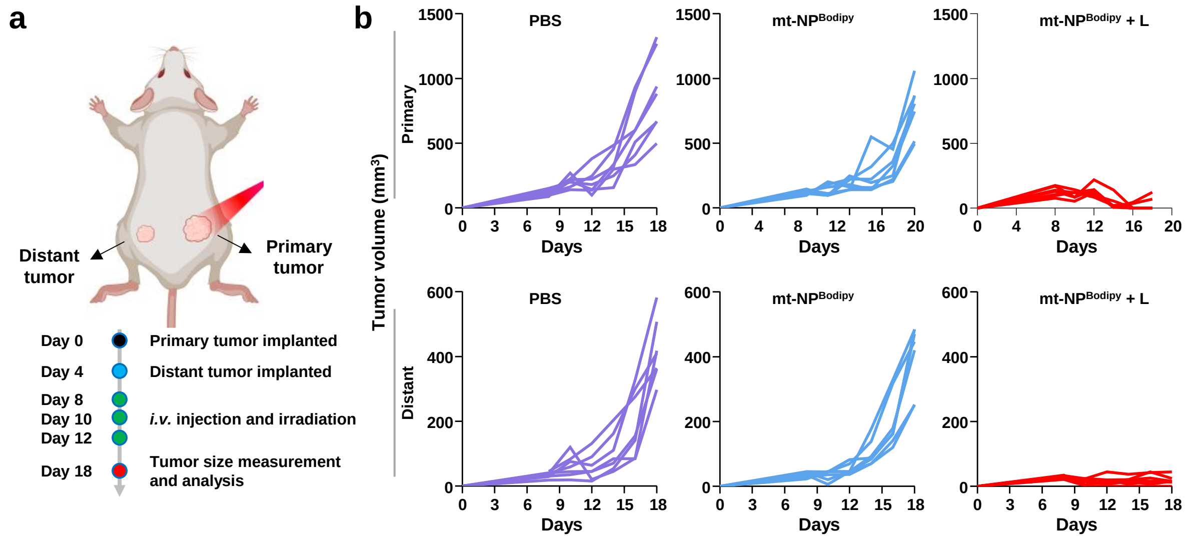

Supplementary Fig. 22. **a** Schematic treatment schedule. Figure created with Biorender.com. **b** Individual and average tumor growth curves of primary and distant tumors (n = 7). This is the data of tumour value each mouse in Fig. 7b,c, and significant differences between mt-NP^{Bodipy} + L and mt-NP^{Bodipy} have been annotated in the corresponding captions (****p < 0.0001). Moreover, the significant differences between mt-NP^{Bodipy} + L and PBS have been annotated in the corresponding captions (****p < 0.0001).

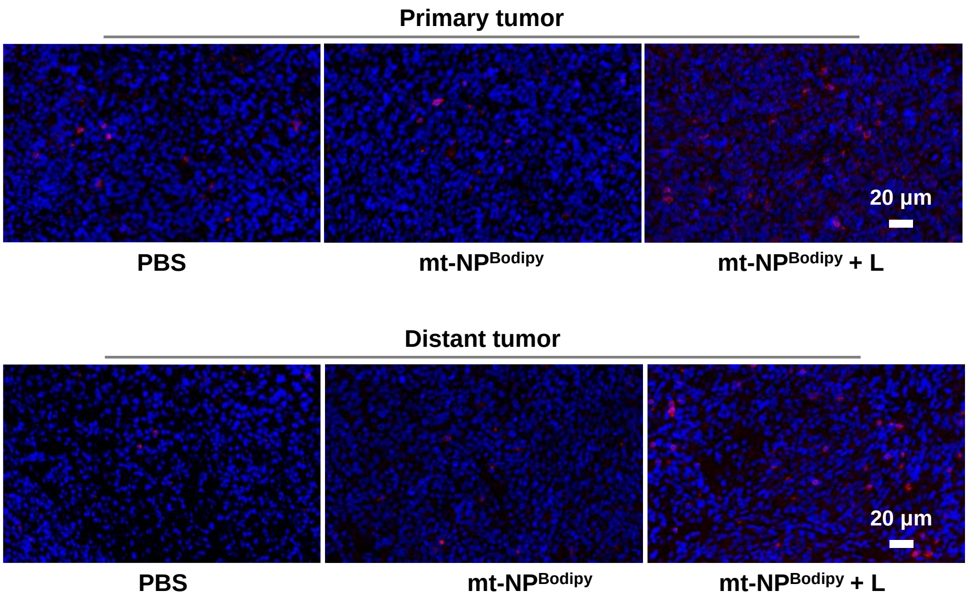

Supplementary Fig. 23. a Representative immunofluorescent staining images of primary tumors and distant tumors. DAPI (blue) and $CD8^+$ (red). Experiment was repeated three times independently with similar results.

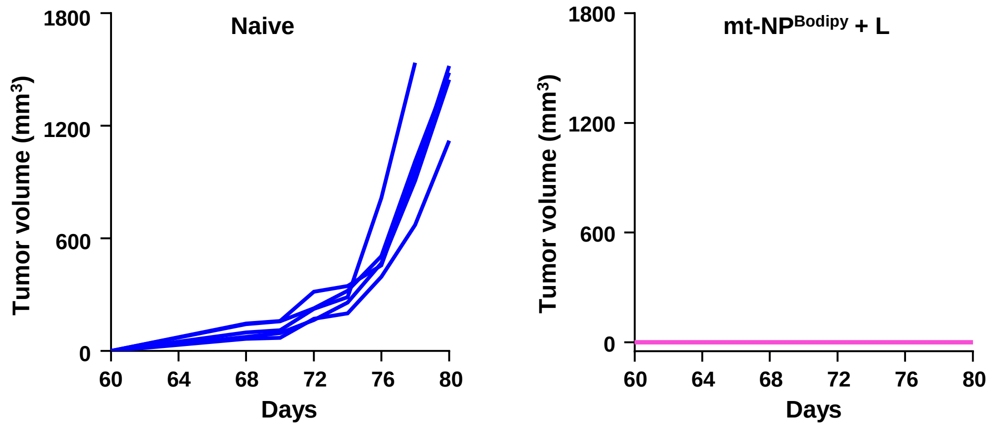

Supplementary Fig. 24. Individual tumor growth curves of the treated mice (n = 5). This is the data of tumour value each mouse in Fig. 7i, and significant differences between mt-NP^{Bodipy} + L and naive have been annotated in the corresponding captions (*****p* < 0.0001).
