# Supplementary material for: Nanoparticles destabilizing the cell membranes triggered by NIR light for cancer imaging and photo-immunotherapy
Source: Nat Commun. 2024 Jul 17;15:6026. doi: 10.1038/s41467-024-50020-w (PMC11255282; doi:10.1038/s41467-024-50020-w)
Supplement: Supplementary file 3 — Reporting Summary [file 41467_2024_50020_MOESM3_ESM.pdf]

nature portfolio

Corresponding author(s): Haihua, Xiao

Last updated by author(s): 21-2-2024

Reporting Summary

Nature Portfolio wishes to improve the reproducibility of the work that we publish. This form provides structure for consistency and transparency in reporting. For further information on Nature Portfolio policies, see our Editorial Policies and the Editorial Policy Checklist.

Statistics

For all statistical analyses, confirm that the following items are present in the figure legend, table legend, main text, or Methods section.

n/a	Confirmed	
☐	☒	The exact sample size (n) for each experimental group/condition, given as a discrete number and unit of measurement
☐	☒	A statement on whether measurements were taken from distinct samples or whether the same sample was measured repeatedly
☐	☒	The statistical test(s) used AND whether they are one- or two-sided<br>*Only common tests should be described solely by name; describe more complex techniques in the Methods section.*
☐	☒	A description of all covariates tested
☐	☒	A description of any assumptions or corrections, such as tests of normality and adjustment for multiple comparisons
☐	☒	A full description of the statistical parameters including central tendency (e.g. means) or other basic estimates (e.g. regression coefficient) AND variation (e.g. standard deviation) or associated estimates of uncertainty (e.g. confidence intervals)
☐	☒	For null hypothesis testing, the test statistic (e.g. F, t, r) with confidence intervals, effect sizes, degrees of freedom and P value noted<br>*Give P values as exact values whenever suitable.*
☒	☐	For Bayesian analysis, information on the choice of priors and Markov chain Monte Carlo settings
☒	☐	For hierarchical and complex designs, identification of the appropriate level for tests and full reporting of outcomes
☒	☐	Estimates of effect sizes (e.g. Cohen's d, Pearson's r), indicating how they were calculated

Our web collection on statistics for biologists contains articles on many of the points above.

Software and code

Policy information about availability of computer code

Data collection	No software was used
Data analysis	Prism 8 (GraphPad Software)

For manuscripts utilizing custom algorithms or software that are central to the research but not yet described in published literature, software must be made available to editors and reviewers. We strongly encourage code deposition in a community repository (e.g. GitHub). See the Nature Portfolio guidelines for submitting code & software for further information.

Data

Policy information about availability of data

All manuscripts must include a data availability statement. This statement should provide the following information, where applicable:

- Accession codes, unique identifiers, or web links for publicly available datasets
- A description of any restrictions on data availability
- For clinical datasets or third party data, please ensure that the statement adheres to our policy

The raw sequence data generated in this study have been deposited in the National Center for Biotechnology Information (NCBI) Sequence Read Archive (SRA) database (Bioproject ID PRJNA976350) under accession code https://www.ncbi.nlm.nih.gov/sra/PRJNA976350. The remaining data are available within the Article, Supplementary Information, or Source Data file. Source data are provided with this paper.

Research involving human participants, their data, or biological material

Policy information about studies with human participants or human data. See also policy information about sex, gender (identity/presentation), and sexual orientation and race, ethnicity and racism.

Reporting on sex and gender	n/a
Reporting on race, ethnicity, or other socially relevant groupings	n/a
Population characteristics	n/a
Recruitment	n/a
Ethics oversight	n/a

Note that full information on the approval of the study protocol must also be provided in the manuscript.

Field-specific reporting

Please select the one below that is the best fit for your research. If you are not sure, read the appropriate sections before making your selection.

☒ Life sciences ☐ Behavioural & social sciences ☐ Ecological, evolutionary & environmental sciences

For a reference copy of the document with all sections, see nature.com/documents/nr-reporting-summary-flat.pdf

Life sciences study design

All studies must disclose on these points even when the disclosure is negative.

Sample size	No statistical methods were used to predetermine the sample sizes. It is impossible to predict the magnitude of experimental variation between animals based on our current knowledge. The group sizes (at least five animals per treatment group) represents the minimum number animals needed to reach statistical significance ($p < 0.05$) between experimental groups.
Data exclusions	No data were excluded.
Replication	Experiment were repeated and experimental findings were reproducible. Details of experimental replicates are given in the figure legends. All reported attempts at replication were successful.
Randomization	All experimental samples or models were allocated randomly to each group.
Blinding	No blinding was done in this study. Most of the studies contained multiple steps (including the material preparation, mouse tumor treatment, and so on) and the scientists must keep careful track of conditions. It would be exceedingly difficult to blind such studies.

Reporting for specific materials, systems and methods

We require information from authors about some types of materials, experimental systems and methods used in many studies. Here, indicate whether each material, system or method listed is relevant to your study. If you are not sure if a list item applies to your research, read the appropriate section before selecting a response.

Materials & experimental systems

n/a	Involved in the study	
☐	☒	Antibodies
☐	☒	Eukaryotic cell lines
☒	☐	Palaeontology and archaeology
☐	☒	Animals and other organisms
☒	☐	Clinical data
☒	☐	Dual use research of concern
☒	☐	Plants

Methods

n/a	Involved in the study	
☒	☐	ChIP-seq
☐	☒	Flow cytometry
☒	☐	MRI-based neuroimaging

Antibodies

Antibodies used	ELISA kits for the mouse cytokines TNF-α (ab208348) and IFN-γ (ab252363) were purchased from Abcam. Anti-mouse antibodies anti-

Antibodies used	CD3-PE (ab22268, 1:200 dilution), anti-CD4-APC (ab252152, 1:200 dilution), anti-CD8-FITC (ab237367, 1:200 dilution), anti-CD11c-PE (ab210309, 1:200 dilution), anti-CD80-FITC (ab18279, 1:200 dilution), anti-CD86-APC (ab218757, 1:200 dilution), anti-F4/80-PE (ab105156, 1:200 dilution), anti-CD62L-APC (ab41459, 1:200 dilution), and anti-CD44-PC5.5 (ab234445, 1:200 dilution) were purchased from Abcam. Anti-mouse antibodies anti-CD206-FITC (E-AB-F1135C, 1:200 dilution) were obtained from Elabscience.
Validation	All antibodies were verified by the supplier and each lot has been quality tested. All validation statements can be found on the respective antibody website:<br>1. ELISA kits for the mouse cytokines TNF-α: https://www.abcam.cn/products/elisa/mouse-tnf-alpha-elisa-kit-ab208348.html<br>2. ELISA kits for the mouse cytokines IFN-γ: https://www.abcam.cn/products/elisa/mouse-ifn-beta-elisa-kit-ab252363.html<br>3. anti-CD3-PE: https://www.abcam.cn/products/primary-antibodies/pe-cd3-antibody-kt3-ab22268.html<br>4. anti-CD4-APC: https://www.abcam.cn/products/primary-antibodies/apc-cd4-antibody-epr20122-ab252152.html<br>5. anti-CD8-FITC: https://www.abcam.cn/products/primary-antibodies/fitc-cd8-alpha-antibody-epr21769-ab237367.html<br>6. anti-CD11c-PE: https://www.abcam.cn/products/primary-antibodies/pe-cd11c-antibody-n418-ab210309.html<br>7. anti-CD80-FITC: https://www.abcam.cn/products/primary-antibodies/fitc-cd80-antibody-mem-233-ab18279.html<br>8. anti-CD86-APC: https://www.abcam.cn/products/primary-antibodies/apc-cd86-antibody-gl-1-gl1-ab218757.html<br>9. anti-F4/80-PE: https://www.abcam.cn/products/primary-antibodies/pe-f480-antibody-cia3-1-ab105156.html<br>10. anti-CD62L-APC: https://www.abcam.cn/products/primary-antibodies/apc-cd62l-antibody-mel-14-ab41459.html<br>11. anti-CD44-PC5.5: https://www.abcam.cn/products/primary-antibodies/percpcy55-cd44-antibody-im7-ab234445.html<br>12. anti-CD206-FITC: https://www.elabscience.cn/p-fitc_anti_mouse_cd206_mmr_antibody_c068c2-e_ab_f1135c

Eukaryotic cell lines

Policy information about cell lines and Sex and Gender in Research

Cell line source(s)	The CT-26 cells lines (IM-M007*T25) were purchased from IMMOCELL (Xiamen, Fujian, China).
Authentication	Identity of the cell lines were frequently checked by their morphological features but have not been authenticated by the short tandem repeat (STR) profiling.
Mycoplasma contamination	The cells were detected every two months to exclude mycoplasma.
Commonly misidentified lines (See ICLAC register)	No commonly misidentified cell lines are used in this study.

Animals and other research organisms

Policy information about studies involving animals; ARRIVE guidelines recommended for reporting animal research, and Sex and Gender in Research

Laboratory animals	BALB/c mice (SPF Biotechnology Co., Ltd. (Beijing, China), 4-5 weeks old, female) were used. Mice were housed in 12h light/12h dark cycle with the temperature maintained between 65-75°F (~18-23°C), ~50% humidity.
Wild animals	The study did not involve wild animals.
Reporting on sex	BALB/c mice (SPF Biotechnology Co., Ltd. (Beijing, China), 4-5 weeks old, female) were used.
Field-collected samples	The study did not involve samples collected from the field.
Ethics oversight	The animals were maintained under pathogen-free conditions and all animal experiments were approved by the Peking University Institutional Animal Care and Use Committee (LA2021316).

Note that full information on the approval of the study protocol must also be provided in the manuscript.

Plants

Seed stocks	*Report on the source of all seed stocks or other plant material used. If applicable, state the seed stock centre and catalogue number. If plant specimens were collected from the field, describe the collection location, date and sampling procedures.*
Novel plant genotypes	*Describe the methods by which all novel plant genotypes were produced. This includes those generated by transgenic approaches, gene editing, chemical/radiation-based mutagenesis and hybridization. For transgenic lines, describe the transformation method, the number of independent lines analyzed and the generation upon which experiments were performed. For gene-edited lines, describe the editor used, the endogenous sequence targeted for editing, the targeting guide RNA sequence (if applicable) and how the editor was applied.*
Authentication	*Describe any authentication procedures for each seed stock used or novel genotype generated. Describe any experiments used to assess the effect of a mutation and, where applicable, how potential secondary effects (e.g. second site T-DNA insertions, mosiacism, off-target gene editing) were examined.*

Flow Cytometry

Plots

Confirm that:

☒ The axis labels state the marker and fluorochrome used (e.g. CD4-FITC).

☒ The axis scales are clearly visible. Include numbers along axes only for bottom left plot of group (a 'group' is an analysis of identical markers).

☒ All plots are contour plots with outliers or pseudocolor plots.

☒ A numerical value for number of cells or percentage (with statistics) is provided.

Methodology

Sample preparation	The obtained tumor tissues, or draining LNDs were used to prepare single-cell suspensions. The single-cell suspensions were further incubated with various antibodies against the immune cells. For the analysis of T cells in the tumor, cells were stained by anti-CD3-PE, anti-CD4-APC, and anti-CD8-FITC. For analyzing DCs in tumors and lymph nodes, cells were stained by anti-CD11c-PE, anti-CD80-FITC, and anti-CD86-APC. For characterizing TAM in tumors, cells were stained by anti-F4/80-PE and anti-CD206-FITC. For analyzing memory T cells in LNDs, cells were stained by anti-CD3-PE, anti-CD8-FITC, anti-CD62L-APC, and anti-CD44-PC5.5. Flow cytometric data acquisition was performed with CytExpert software, and the data were processed using FlowJo software.
Instrument	flow cytometer (BD Biosciences, USA)
Software	All statistical calculations were carried out with Prism 8 (GraphPad Software).
Cell population abundance	No sorting was performed.
Gating strategy	Generally, cells were first gated on FSC/SSC. Singlet cells were usually gated using FSC-H and FSC-A. Surface antigen gating was performed on the live cell population.

☒ Tick this box to confirm that a figure exemplifying the gating strategy is provided in the Supplementary Information.
